# Emerging echinocandin-resistant *Candida albicans* and *glabrata* in Switzerland

**DOI:** 10.1007/s15010-020-01475-8

**Published:** 2020-07-13

**Authors:** A. T. Coste, A. Kritikos, J. Li, N. Khanna, D. Goldenberger, C. Garzoni, C. Zehnder, K. Boggian, D. Neofytos, A. Riat, D. Bachmann, D. Sanglard, F. Lamoth, The Fungal Infection Network of Switzerland (FUNGINOS), F. Lamoth, N. Khanna, K. Boggian, D. Sanglard

**Affiliations:** 1 Institute of Microbiology, Lausanne University Hospital and University of Lausanne, Lausanne, Switzerland; 2 Service of Infectious Diseases, Centre Hospitalier Universitaire Vaudois, Lausanne University Hospital and University of Lausanne, Rue du Bugnon 46, 1011 Lausanne, Switzerland; 3 Division of Infectious Diseases and Hospital Epidemiology, University and University Hospital of Basel, Basel, Switzerland; 4 Division of Clinical Bacteriology and Mycology, University and University Hospital of Basel, Basel, Switzerland; 5 Clinica Luganese Moncucco, Lugano, Switzerland; 6 SYNLAB Suisse SA, Bioggio, Switzerland; 7 Division of Infectious Diseases and Hospital Epidemiology, Kantonsspital St.Gallen, St.Gallen, Switzerland; 8 Service of Infectious Diseases, Geneva University Hospitals, Geneva, Switzerland; 9 Service of Laboratory Medicine, Geneva University Hospitals, Geneva, Switzerland

## Abstract

Echinocandins represent the first-line therapy of candidemia. Echinocandin resistance among *Candida* spp. is mainly due to acquired *FKS* mutations. In this study, we report the emergence of *FKS*-mutant *Candida albicans*/*glabrata* in Switzerland and provide the microbiological and clinical characteristics of 9 candidemic episodes. All patients were previously exposed to echinocandins (median 26 days; range 15–77). Five patients received initial echinocandin therapy with persistent candidemia in 4 of them. Overall mortality was 33%.

## Introduction

The pathogenic yeasts *Candida* spp. are an important cause of nosocomial bloodstream infections, which are associated with high mortality rates [1]. *Candida albicans* represents the most frequent cause of candidemia, but a progressive epidemiological shift towards more resistant non-*albicans Candida* spp. (e.g., *Candida glabrata*) is reported all over the world [1]. In this context, echinocandins (e.g., anidulafungin, caspofungin and micafungin) have become the first-line antifungal therapy because of their better efficacy and broader antifungal spectrum against *Candida* spp. compared to azoles [2]. However, increased use of echinocandins has been associated with the emergence of resistance to these drugs, which affects particularly *C. albicans* and *C. glabrata* [3]. Echinocandin resistance in *Candida* spp. results from well-defined mutations in hotspot regions of the *FKS* gene that encodes for the 1,3-beta-d-beta-glucan synthase, the target of echinocandins [4]. These mutations usually result in pan-echinocandin resistance and affect mainly *C. glabrata* (prevalence range 2–13%) and more rarely *C. albicans* (< 1%) [3, 5–9]. Previous echinocandin exposure has been identified as the main risk factor and was observed in most cases [3, 5]. Because of the limited therapeutic alternatives, in particular for *C. glabrata* candidemia, mortality rates are high (60%) [5]. In Switzerland, a recent nationwide survey of candidemia (2004–2013) reported a very low rate of *FKS*-mutant *C. albicans* and *C. glabrata* (2 out of 1624 isolates, 0.12%) [10]. However, several echinocandin-resistant isolates harbouring various types of *FKS* mutations have been reported from different centers since that time. The aim of this study was to characterize these strains and to report the clinical characteristics and outcome of their associated candidemic episodes.

## Materials and methods

### Patients and data collection

Clinical isolates of candidemic *C. albicans* and *C. glabrata* that were non-susceptible to micafungin and/or anidulafungin according to the clinical breakpoints defined by the Clinical and Laboratory Standards Institute (CLSI) [11] were retrospectively identified by (i) screening of a nationwide survey of candidemia by the Fungal Infection Network of Switzerland (FUNGINOS) conducted from 2004 to 2013 in 25 university and university-affiliated medical centers of Switzerland, and (ii) a call to the microbiologists of the university hospitals to screen and send their candidemic *Candida* spp. isolates with phenotypic echinocandin resistance to the reference laboratory (Lausanne University Hospital) for the period 2013–2019. Demographic characteristics and clinical data were collected via a standard clinical report form (CRF) including underlying conditions, risk factors for candidemia, previous courses of antifungal therapy within the last 3 months, clinical characteristics, treatment and outcome of *Candida* infection.

### Antifungal susceptibility testing and sequencing

All *Candida* isolates were handled in our reference laboratory. Minimum inhibitory concentrations (MIC) of anidulafungin, micafungin, caspofungin and fluconazole were retested in duplicates by microbroth dilution method according to the protocol M27 (4th edition) of the CLSI [11]. Susceptibility (*S*), dose-dependent susceptibility (SDD) or resistance (*R*) were defined according to the CLSI breakpoints, which are for anidulafungin and caspofungin (in µg/mL): *C. albicans* *S* ≤ 0.25, *R* ≥ 1, *C. glabrata* *S* ≤ 0.12, *R* ≥ 0.5, for micafungin: *C. albicans* *S* ≤ 0.25, *R* ≥ 1, *C. glabrata* *S* ≤ 0.06, *R* ≥ 0.25, and for fluconazole: *C. albicans* *S* ≤ 2, *R* ≥ 8, *C. glabrata* SDD ≤ 32, *R* ≥ 64. Isolates for which MICs were confirmed to be above the susceptibility clinical breakpoints (i.e., classified as non-susceptible according to the CLSI breakpoints) for micafungin and/or anidulafungin were selected for genotypic characterization of *FKS* genes (of note, isolated elevated caspofungin MIC was not considered as a reliable predictor of *FKS* mutations on the basis of previous publications [10, 12]). PCR and sequencing of the hotspot regions (HS) of the *FKS* genes (HS1 and HS2 of *FKS1* for *C. albicans* and *C. glabrata* and HS1 and HS2 of *FKS2* for *C. glabrata*) were performed using the primers previously described [10].

### Ethical statement

This study was approved by the Swiss ethics committee on research involving humans for retrospective use of clinical data (Swissethics reference # 2019-00484_1904).

## Results

A total of 9 cases of candidemia due to echinocandin-resistant *Candida* spp. (4 *C. albicans* and 5 *C. glabrata*) were identified. Two cases with confirmed *FKS* mutations were obtained from the FUNGINOS nationwide survey from 2004 to 2013. Seven additional cases were reported from 4 hospitals from 2013 to 2019. In total, 5 of the 9 cases were observed within the last 2 years (2018–2019). For 4 cases, a susceptible isolate of the same *Candida* species was obtained from previous blood cultures (4–19 days before the candidemic episode attributed to the resistant isolate). MIC of anidulafungin, micafungin, caspofungin and fluconazole, as well as results of *FKS* hotspots sequencing for all the 9 cases (13 isolates, including the 9 echinocandin-resistant strains and the 4 previously susceptible strains) are shown in Table 1. The most frequent mutations were S645P (*n* = 3) for *C. albicans* and S663P in *FKS2* (*n* = 3) for *C. glabrata*. The 3 remaining echinocandin-resistant isolates were one *C. albicans* with R1361G mutation and two *C. glabrata* isolates with S629P (*FKS1*) and F659_(*FKS2*) mutations, respectively. All 9 isolates were classified as non-susceptible to all three echinocandins according to CLSI breakpoints. One of the four echinocandin-resistant *C. albicans* isolates and all *C. glabrata* isolates were classified as susceptible dose-dependent to fluconazole. Absence of *FKS* mutations was confirmed for the 4 susceptible isolates obtained from the previous candidemic episodes.

**Table 1:** Results of antifungal susceptibility testing and *FKS* sequencing of *C. albicans* and *C. glabrata* from candidemic episodes

Cases	Species	Episode	MIC µg/mL (CLSI classification)*				FKS hotspot mutations**			
			AND	MCF	CSP	FLC	*FKS1*		*FKS2*	
							HS1	HS2	HS1	HS2
1	*C. albicans*	1st	0.25 (S)	0.125 (S)	0.5 (I)	4 (SDD)	–	–	na	
		**2**nd	**2 (R)**	**2 (R)**	**4 (R)**	**4 (SDD)**	**S645P**	–		
2	*C. albicans*	**1**st	**2 (R)**	**4 (R)**	**8 (R)**	**2 (S)**	**S645P**	–		
3	*C. albicans*	**1**st	**2 (R)**	**4 (R)**	** > 16 (R)**	** < 0.5 (S)**	**S645P**	–		
4	*C. albicans*	**1**st	**2 (R)**	**1 (R)**	**1 (R)**	** < 0.5 (S)**	–	**R1361G**		
5	*C. glabrata*	**1**st	**2 (R)**	**4 (R)**	** > 16 (R)**	**4 (SDD)**	–	–	**S663P**	–
6	*C. glabrata*	**1**st	**4 (R)**	**4 (R)**	**16 (R)**	**4 (SDD)**	–	–	**F659_**	–
7	*C. glabrata*	1st	0.25 (I)	0.06 (S)	0.25 (I)	1 (SDD)	–	–	–	–
		**2**nd	**4 (R)**	**4 (R)**	** > 16 (R)**	**1 (SDD)**	–	–	**S663P**	–
8	*C. glabrata*	1st	0.12 (S)	0.015 (S)	0.06 (S)	8 (SDD)	–	–	–	–
		**2**nd	**4 (R)**	**4 (R)**	** > 16 (R)**	**4 (SDD)**			**S663P**	
9	*C. glabrata*	1st	0.015 (S)	0.015 (S)	0.03 (S)	4 (SDD)	–	–	–	–
		**2**nd	**0.25 (I)**	**4 (R)**	**16 (R)**	**8 (SDD)**	**S629P**	–	–	–

*MIC* minimal inhibitory concentration, *CLSI* Clinical and Laboratory Standards Institute, *AND* anidulafungin, *MCF* micafungin, *CSP* caspofungin, *FLC* fluconazole, *HS* hotspot, *R* resistant, *I* intermediate, *S* susceptible, *SDD* susceptible dose-dependent

^*^CLSI breakpoints [µg/mL]: anidulafungin and caspofungin: *C. albicans S* ≤ 0.25, *R* ≥ 1, *C. glabrata S* ≤ 0.12, *R* ≥ 0.5; Micafungin: *C. albicans S* ≤ 0.25, *R* ≥ * R* ≥ 1, *C. glabrata S* ≤ 0.06, *R* ≥ 0.25; Fluconazole: *C. albicans S* ≤ 2, *R* ≥ 8, *C. glabrata* SDD ≤ 32, *R* ≥ 64

^**^All the indicated non-synonymous mutations were already demonstrated as responsible for echinocandin resistance. In bold: resistant isolates harboring *FKS* mutations. If available, sequencing and antifungal susceptibility testing results of previous candidemic episodes are shown (not in bold)

Clinical characteristics of the 9 cases are shown in Table 2. All patients have been exposed to echinocandins before the candidemic episode with the resistant isolate (median duration: 26 days, range 15–77). Five episodes were breakthrough candidemia (i.e., occurring while echinocandin treatment was ongoing). Five cases had a deep focus of infection (3 abdominal and 2 urinary), while candidemia was primary or originating from a catheter in 4 cases. Five patients received initial echinocandin therapy (duration 7–18 days) with persistent candidemia (duration between first and last positive blood cultures: 5–40 days) in 4 of them. In these cases, candidemia was cleared after switch to another antifungal drug (liposomal amphotericin B in most cases). Three of the nine patients died within 6 weeks from the candidemia and death was at least partially attributed to candidemia (i.e., active disease at time of death) in two cases.

**Table 2:** Clinical characteristics of echinocandin-resistant *C. albicans* and *C. glabrata* candidemic episodes

Case (year)	Underlying diseases	*Candida* species	Prior echinocandin exposure, days (drug)	Origin of candidemia	Duration of candidemia (days first-last positive culture)	Treatment	Outcome (days from diagnosis) role of candidemia
1 (2014)	Male, 78 y.o Prostatic cancer Urinary tract surgery	*C. albicans*	20 (CSP)	Urinary	5	Surgery Catheter removal FLC (17 days)	Died (day 23) Partial
2 (2018)	Male, 31 y.o Myeloablative chemotherapy for acute promyelocytic leukemia	*C. albicans*	15 (CSP)	Catheter	0	L-AMB (1 day)	Died (day 2) Major
3 (2018)	Male, 70 y.o Secondary peritonitis after vascular graft implantation	*C. albicans*	27 (CSP)	Abdominal	0	Surgery FLC (nd)	Died (day 42) No
4 (2013)	Female, 74 y.o Abdominal surgery	*C. albicans*	61 (AND/CSP)	Abdominal	14	CSP (7 days), then L-AMB (28 days) Catheter removal	Cured
5 (2008)	Male, 26 y.o Mitochondrial encephalomyopathy Abdominal surgery Allogeneic HSCT	*C. glabrata*	25 (CSP)	Catheter	0	CSP (14 days) Catheter removal	Cured
6 (2015)	Male, 60 y.o Acute myeloid leukemia Allogeneic HSCT	*C. glabrata*	-	Catheter	40	CSP (18 days), then L-AMB (43 days) Catheter removal	Cured
7 (2019)	Male, 84 y.o Gallbladder surgery	*C. glabrata*	18 (CSP)	Urinary	5	CSP (7 days), then L-AMB (16 days), then FLC (20 days)	Cured
8 (2019)	Female, 52 y.o Abdominal surgery, secondary peritonitis	*C. glabrata*	77 (CSP)	Abdominal	2	Catheter removal L-AMB (13 days)	Cured
9 (2019)	Female, 25 y.o Abdominal surgery, tertiary peritonitis Renal transplantation	*C. glabrata*	47 (CSP/AND)	Catheter	21	Catheter removal AND (17 days), then L-AMB (7 days), then FLC (22 days)	Cured

*y.o.* year old, *HSCT* hematopoietic stem cell transplantation, *AND* Anidulafungin, *CSP* Caspofungin, *L-AMB* liposomal amphotericin B, *FLC* fluconazole

## Discussion

In this study, we described the microbiological and clinical characteristics of nine episodes of candidemia due to *FKS*-mutant *C. albicans*/*glabrata* that were observed in Switzerland between 2004 and 2019. The actual prevalence of these *FKS* mutations among *C. albicans*/*glabrata* candidemic episodes was assessed for the period 2004–2013 via a national surveillance program of the Fungal Infection Network of Switzerland (FUNGINOS) collecting all candidemic isolates from 25 Swiss medical centers (including all university and most university-affiliated hospitals) [10]. Only 2 *FKS*-mutant isolates were recovered from this 10-year period (prevalence 0.12%). We could not assess the prevalence for the period 20,014–2019, where only isolates with phenotypic resistance from a limited subset of university hospitals were sent to our laboratory for *FKS* sequencing. However, the fact that we identified 7 *FKS*-mutant *C. albicans*/*glabrata* from 4 different Swiss centers over this 6-year period suggests an increase of the rate of *FKS*-mutant echinocandin resistant isolates. The incidence of candidemia remained relatively stable in Switzerland over the last decade according to our national surveillance systems (unpublished data), but we have observed a constant increase of echinocandin consumption since 2004, as previously reported [10]. Echinocandin pre-exposure was previously identified as the main risk factor for *FKS* mutations [3, 5]. In our study, all patients received previous echinocandin treatment for > 15 days, which is in keeping with previous reports (median 3–4 weeks) [3, 5]. Caspofungin is the most used echinocandin drug in Switzerland and a recent publication suggests that it is associated with a higher risk of inducing *FKS* mutations in comparison to other echinocandins [13]. Other risk factors of acquired *FKS* mutations have been suggested, such as the existence of hidden reservoirs or uncontrolled sources of infection [14], which was probably the case for more than half (5/9) patients of our series with complicated intra-abdominal candidiasis or persistent urinary tract colonization. Among the 5 patients who received initial echinocandin therapy fo* R* ≥ 7 days, 4 failed to respond (i.e., persistent candidemia under echinocandin therapy) and ultimately survived after switch for another antifungal drug. The patient who responded to echinocandin therapy despite high MIC values had an intravascular catheter infection and was possibly cured by catheter removal only. The cases for which echinocandin therapy was initially continued, despite resistance to this antifungal drug class, were mainly patients with severe comorbidities (including renal dysfunction) who were infected with *C. glabrata*, a species exhibiting some degree of natural resistance to azoles. This outlines the difficulty to treat such infections because of the lack of therapeutic alternatives. Amphotericin B formulations often remain the unique option and their use could be limited by renal toxicity. However, our results clearly suggest that echinocandin therapy should not be continued against these *FKS*-mutant *Candida* species, even in those with moderate MIC elevation (e.g., 2–4 µg/mL), and is associated with failure to therapy. Overall mortality was 33%, which is in the usual ranges of mortality rates that have been reported for candidemia in general [1].

*FKS* mutations observed in our study were diverse, but predominantly S645P in *C. albicans* and S663P (*FKS2*) in *C. glabrata*. All *FKS*-mutant isolates of our study exhibited echinocandin MICs that were classified as non-susceptible according to the CLSI breakpoints. However, MIC values varied between 0.25 and  > 16 µg/mL with caspofungin MIC being usually higher compared to anidulafungin or micafungin MICs.

Echinocandin resistance among *Candida* spp. remains rare. Previous observations are limited to small case-series (7–25 cases) with sometimes incomplete clinical data and mainly reported from the North American continent [3, 5, 6, 8, 9]. The present report of these 9 cases suggests that echinocandin resistance due to acquired *FKS* mutations is an emerging concern in Europe. These cases were associated with failure of echinocandin therapy, which is particularly concerning for *C. glabrata* due to the limited therapeutic options. Echinocandin resistance should be suspected in patients with previous echinocandin exposure (> 15 days), particularly in the presence of hidden or uncontrolled reservoirs, such as abdominal candidiasis or urinary tract colonization.

## Availability of data and material

The sequences of *FKS* hotspots regions of all *Candida* isolates of this study have been deposited at the National Center for Biotechnology Information (NCBI) under Accession Numbers MT396587 to MT396628.
